# Majorization problems for two subclasses of analytic functions connected with the Liu–Owa integral operator and exponential function

**DOI:** 10.1186/s13660-018-1865-x

**Published:** 2018-10-11

**Authors:** Huo Tang, Guantie Deng

**Affiliations:** 1 School of Mathematics and Statistics, Chifeng University, Chifeng, People’s Republic of China; 2 School of Mathematical Sciences, Beijing Normal University, Beijing, People’s Republic of China

**Keywords:** 30C45, 30C80, Analytic function, Subordination, Majorization, Exponential function, Liu–Owa integral operator

## Abstract

In the present paper, we investigate majorization properties for the class $M_{\beta}^{\alpha}(p,\gamma)$ of uniformly starlike functions and the class $N_{\beta}^{\alpha}(p,\theta)$ of spiral-like functions related to an exponential function, which are defined through the Liu–Owa integral operator $Q_{\beta,p}^{\alpha}$ given by (1.5). Also, some special cases of our main results in a form of corollaries are shown.

## Introduction and definitions

Let $\mathbb{C}$ be a complex plane and assume that $\mathcal{A}_{p}$ denotes the class of analytic and *p*-valent functions of the form

1.1 $$ f(z)=z^{p}+\sum_{k=1}^{\infty}a_{k+p}z^{k+p} \quad \bigl(p\in\mathbb{N}=\{1,2,\ldots \}\bigr) $$

in the open unit disk

$$\mathbb{U}=\bigl\{ z:z\in\mathbb{C} \text{ and } \vert z \vert < 1\bigr\} . $$

Specially, for $p=1$, we write $\mathcal{A}:=\mathcal{A}_{1}$.

In 1967, MacGregor [22] introduced the notion of majorization as follows.

### Definition 1.1

Let *f* and *g* be analytic in $\mathbb{U}$. We say that *f* is majorized by *g* in $\mathbb{U}$ and write

$$f(z)\ll g(z)\quad (z\in\mathbb{U}), $$

if there exists a function $\varphi(z)$, analytic in $\mathbb{U}$, satisfying

1.2 $$ \bigl\vert \varphi(z) \bigr\vert \leq1\quad \text{and}\quad f(z)= \varphi(z)g(z)\quad (z\in\mathbb{U}). $$

In 1970, Roberston [28] gave the concept of quasi-subordination as follows.

### Definition 1.2

For two analytic functions *f* and *g* in $\mathbb{U}$, we say that *f* is quasi-subordinate to *g* in $\mathbb {U}$ and write

$$f(z)\prec_{q} g(z)\quad (z\in\mathbb{U}), $$

if there exist two analytic functions $\varphi(z)$ and $\omega(z)$ in $\mathbb{U}$ such that $\frac{f(z)}{\varphi(z)}$ is analytic in $\mathbb {U}$ and

$$\bigl\vert \varphi(z) \bigr\vert \leq1, \qquad \omega(0)=0 \quad \text{and} \quad \bigl\vert \omega(z) \bigr\vert \leq \vert z \vert < 1\quad (z\in \mathbb{U}), $$

satisfying

1.3 $$ f(z)=\varphi(z)g\bigl(\omega(z)\bigr)\quad (z\in\mathbb{U}). $$

### Remark 1.3

(i) For $\varphi(z)\equiv1$ in (1.3), we have
  $$f(z)=g\bigl(\omega(z)\bigr)\quad (z\in\mathbb{U}) $$
  and say that *f* is subordinate to *g* in $\mathbb{U}$, denoted by (see [29])
  $$f(z)\prec g(z)\quad (z\in\mathbb{U}). $$
(ii) For $\omega(z)=z$ in (1.3), the quasi-subordination (1.3) becomes the majorization (1.2).

In 1991, Ma and Minda [21] introduced the following function class $S^{*}(\phi)$, which is defined by using the above subordination principle:

$$S^{*}(\phi):= \biggl\{ f\in\mathcal{A}:\frac{zf'(z)}{f(z)}\prec\phi(z)\ (z\in \mathbb{U}) \biggr\} , $$

where $\phi(z)$ is analytic and univalent in $\mathbb{U}$ and for which $\phi(\mathbb{U})$ is convex with $\phi(0)=1$ and $\Re(\phi(z))>0$ for $z\in\mathbb{U}$.

We notice that, for choosing a suitable function $\phi(z)$, the class $S^{*}(\phi)$ reduces to one of the well-known classes of functions. For instance:

(i) If we take
  $$\phi(z)=\frac{1+Az}{1+Bz}\quad (-1\leq B< A\leq1; z\in\mathbb{U}), $$
  then we obtain the class
  $$S^{*}(A,B):= \biggl\{ f\in\mathcal{A}:\frac{zf'(z)}{f(z)}\prec\frac {1+Az}{1+Bz}\ (-1 \leq B< A\leq1; z\in\mathbb{U}) \biggr\} , $$
  which was introduced by Janowski [16]. As a special case, for $A=1-2\alpha$ and $B=-1$, we have the class $S^{*}(1-2\alpha ,-1)=S^{*}(\alpha)$ of starlike functions of order *α* ($0\leq\alpha <1$). Further, for $A=1$ and $B=-1$, we have the familiar class $S^{*}(1,-1)=S^{*}$ of starlike functions in $\mathbb{U}$.
(ii) If we put
  $$\phi(z)=e^{z}\quad (z\in\mathbb{U}), $$
  then we get the class
  $$S_{e}^{*}:= \biggl\{ f\in\mathcal{A}:\frac{zf'(z)}{f(z)}\prec e^{z}\ (z\in \mathbb{U}) \biggr\} , $$
  which was introduced and investigated by Mendiratta et al. [23] and implies that
  1.4 $$ f\in S_{e}^{*}\quad \Longleftrightarrow\quad \biggl\vert \log \frac{zf'(z)}{f(z)} \biggr\vert < 1\quad (z\in\mathbb{U}). $$

In 2004, Liu and Owa [20] (see also [4–9, 32]) introduced the integral operator $Q_{\beta,p}^{\alpha}: \mathcal {A}_{p}\longrightarrow\mathcal{A}_{p}$ as follows:

1.5 $$ Q_{\beta,p}^{\alpha}f(z)=\left ( \textstyle\begin{array}{@{}c@{}} p+\alpha+\beta-1\\ p+\beta-1 \end{array}\displaystyle \right )\frac{\alpha}{z^{\beta}} \int_{0}^{z} \biggl(1-\frac{t}{z} \biggr)^{\alpha -1}t^{\beta-1}f(t)\,dt\quad (\alpha>0; \beta>-1; p\in \mathbb{N}) $$

and

$$Q_{\beta,p}^{0} f(z)=f(z)\quad (\alpha=0; \beta>-1). $$

If the function $f\in\mathcal{A}_{p}$ given by (1.1), then from (1.5) we show that

1.6 $$\begin{aligned}& Q_{\beta,p}^{\alpha}f(z)=z^{p}+\frac{\Gamma(\alpha+\beta+p)}{\Gamma(\beta +p)}\sum _{k=1}^{\infty} \frac{\Gamma(\beta+p+k)}{\Gamma(\alpha+\beta+p+k)}a_{k+p}z^{k+p} \\& \quad (\alpha \geq0; \beta>-1; p\in\mathbb{N}). \end{aligned}$$

Also, we easily find the relationship, from (1.6), that (see [20])

1.7 $$ z \bigl(Q_{\beta,p}^{\alpha}f(z) \bigr)'=(\alpha+ \beta+p-1)Q_{\beta ,p}^{\alpha-1} f(z)-(\alpha+\beta-1)Q_{\beta,p}^{\alpha}f(z). $$

On the other hand, we observe that

(i) for $p=1$, we get the Jung–Kim–Srivastava integral operator $Q_{\beta}^{\alpha}:=Q_{\beta,1}^{\alpha}$ (see [17]; also see [3, 11]);
(ii) for $\alpha=1$ and $\beta=\delta$, we obtain the generalized Libera operator $J_{\delta,p}:=Q_{\delta,p}^{1}$, which is presented as follows (see [10]; see also [19, 25]):
  1.8 $$ J_{\delta,p}(f) (z):=Q_{\delta,p}^{1} f(z)=\frac{\delta+p}{z^{\delta}} \int _{0}^{z}t^{\delta-1}f(t)\,dt \quad ( \delta>-p; p\in\mathbb{N}). $$

Inspired by the above class $S_{e}^{*}$, we now use the Liu–Owa integral operator $Q_{\beta,p}^{\alpha}$ to define the following two subclasses $M_{\beta}^{\alpha}(p,\gamma)$ and $N_{\beta}^{\alpha}(p,\theta)$ of functions $f\in\mathcal{A}_{p}$.

### Definition 1.4

Let $p\in\mathbb{N}$; $\alpha\geq0$; $\beta>-1$ and $\gamma\geq0$. A function $f\in\mathcal{A}_{p}$ belongs to the class $M_{\beta}^{\alpha}(p,\gamma)$ of uniformly starlike functions, related to exponential function, if and only if

1.9 $$ \biggl[ \biggl(\frac{z(Q_{\beta,p}^{\alpha}f(z))'}{Q_{\beta,p}^{\alpha}f(z)}+1-p \biggr)-\gamma \biggl\vert \frac{z(Q_{\beta,p}^{\alpha}f(z))'}{Q_{\beta ,p}^{\alpha}f(z)}-p \biggr\vert \biggr] \prec e^{z}. $$

### Remark 1.5

(i) For $p=1$ in (1.9), we have the function class
  $$M_{\beta}^{\alpha}(\gamma):=M_{\beta}^{\alpha}(1, \gamma)= \biggl\{ f\in \mathcal{A}: \biggl[\frac{z(Q_{\beta}^{\alpha}f(z))'}{Q_{\beta}^{\alpha}f(z)}-\gamma \biggl\vert \frac{z(Q_{\beta}^{\alpha}f(z))'}{Q_{\beta}^{\alpha}f(z)}-1 \biggr\vert \biggr] \prec e^{z}\ (\gamma\geq0) \biggr\} . $$
(ii) For $\gamma=0$ in (1.9), we get the function class
  $$M_{\beta}^{\alpha}(p):=M_{\beta}^{\alpha}(p,0)= \biggl\{ f\in\mathcal {A}_{p}: \frac{z(Q_{\beta,p}^{\alpha}f(z))'}{Q_{\beta,p}^{\alpha}f(z)}\prec \bigl(e^{z}+p-1 \bigr)\ (p\in\mathbb{N}) \biggr\} . $$
(iii) Further, for $\gamma=p-1=0$ in (1.9), we obtain the function class
  $$M_{\beta}^{\alpha}:=M_{\beta}^{\alpha}(1,0)= \biggl\{ f \in\mathcal{A}: \frac{z(Q_{\beta}^{\alpha}f(z))'}{Q_{\beta}^{\alpha}f(z)}\prec e^{z} \biggr\} . $$

### Definition 1.6

Let $p\in\mathbb{N}$; $\alpha\geq0$; $\beta>-1$ and $-\frac{\pi}{2}<\theta<\frac{\pi}{2}$. A function $f\in\mathcal {A}_{p}$ belongs to the class $N_{\beta}^{\alpha}(p,\theta)$ of spiral-like functions, related to an exponential function, if and only if

1.10 $$ e^{i\theta} \biggl(\frac{z(Q_{\beta,p}^{\alpha}f(z))'}{Q_{\beta,p}^{\alpha}f(z)} \biggr)\prec e^{z}\cos \theta+i\sin\theta. $$

### Remark 1.7

(i) For $p=1$ in (1.10), we obtain the function class
  $$\begin{aligned} N_{\beta}^{\alpha}(\theta)&:=N_{\beta}^{\alpha}(1, \theta) \\ &= \biggl\{ f\in \mathcal{A}: e^{i\theta} \biggl(\frac{z(Q_{\beta}^{\alpha}f(z))'}{Q_{\beta }^{\alpha}f(z)} \biggr)\prec e^{z}\cos\theta+i\sin\theta\ \biggl(-\frac{\pi }{2}< \theta< \frac{\pi}{2}\biggr) \biggr\} . \end{aligned}$$
(ii) For $\theta=0$ in (1.10), we have the function class
  $$N_{\beta}^{\alpha}(p):=N_{\beta}^{\alpha}(p,0)= \biggl\{ f\in\mathcal {A}_{p}: \frac{z(Q_{\beta,p}^{\alpha}f(z))'}{Q_{\beta,p}^{\alpha}f(z)}\prec e^{z}\ (p\in \mathbb{N}) \biggr\} . $$
(iii) Further, for $\theta=p-1=0$ in (1.10), we get the function class $M_{\beta}^{\alpha}=N_{\beta}^{\alpha}:=N_{\beta}^{\alpha }(1,0)$.

A majorization problem for the normalized class of starlike functions has been investigated by MacGregor [22] and Altintas et al. [1] (see also [2]). Recently, many researchers have studied several majorization problems for univalent and multivalent functions or meromorphic and multivalent meromorphic functions, which are all subordinate to certain function $\phi(z)=\frac{1+Az}{1+Bz}$ ($-1\leq B< A\leq1$), involving various different operators; the interested reader can, for example, see [13–15, 18, 26, 27, 30, 31, 33]. However, we note that there is no article dealing with the above-mentioned problems for functions which are subordinate to $\phi (z)=e^{z}$. Hence, in the present paper, we investigate the problems of majorization of the classes $M_{\beta}^{\alpha}(p,\gamma)$ and $N_{\beta }^{\alpha}(p,\theta)$ defined by the Liu–Owa integral operator $Q_{\beta,p}^{\alpha}$ given by (1.5), which are related to an exponential function.

## Majorization problem for the class $M_{\beta}^{\alpha}(p,\gamma )$

Firstly, we give and prove majorization property for the class $M_{\beta}^{\alpha}(p,\gamma)$.

### Theorem 2.1

*Let the function* $f\in\mathcal{A}_{p}$ *and suppose that* $g\in M_{\beta}^{\alpha}(p,\gamma)$ *with* $|\alpha+\beta +p-2|\geq\gamma(\alpha+\beta+p-1)+e$. *If* $Q_{\beta,p}^{\alpha}f(z)$ *is majorized by* $Q_{\beta,p}^{\alpha}g(z)$ *in* $\mathbb{U}$, *that is*,

$$Q_{\beta,p}^{\alpha}f(z)\ll Q_{\beta,p}^{\alpha}g(z) \quad (z\in\mathbb{U}), $$

*then*, *for* $|z|\leq r_{1}$, *we have*

$$\bigl\vert Q_{\beta,p}^{\alpha-1}f(z) \bigr\vert \leq \bigl\vert Q_{\beta,p}^{\alpha-1}g(z) \bigr\vert , $$

*where* $r_{1}=r_{1}(p,\alpha,\beta,\gamma)$ *is the smallest positive root of the equation*

2.1 $$\begin{aligned}& r^{2}e^{r}- \bigl[|\alpha+\beta+p-2|-\gamma(\alpha+ \beta+p-1) \bigr]r^{2} -e^{r}-2(1+\gamma)r \\& \quad {}+|\alpha+\beta+p-2|-\gamma(\alpha+\beta+p-1)=0\quad (p\in\mathbb{N}; \alpha\geq0; \beta>-1; \gamma\geq0). \end{aligned}$$

### Proof

Since $g\in M_{\beta}^{\alpha}(p,\gamma)$, then, from (1.9) and the subordination relationship, we get

2.2 $$ \biggl[ \biggl(\frac{z(Q_{\beta,p}^{\alpha}g(z))'}{Q_{\beta,p}^{\alpha}g(z)}+1-p \biggr)-\gamma \biggl\vert \frac{z(Q_{\beta,p}^{\alpha}g(z))'}{Q_{\beta ,p}^{\alpha}g(z)}-p \biggr\vert \biggr]=e^{\omega(z)}, $$

where $\omega(z)=c_{1}z+c_{2}z^{2}+\cdots$ is bounded and analytic in $\mathbb {U}$, satisfying (see, for details, Goodman [12])

2.3 $$ \omega(0)=0 \quad \text{and} \quad \bigl\vert \omega(z) \bigr\vert \leq|z| \quad (z\in\mathbb{U}). $$

Letting

2.4 $$ \varpi=\frac{z(Q_{\beta,p}^{\alpha}g(z))'}{Q_{\beta,p}^{\alpha}g(z)}+1-p $$

in (2.2), we have

$$\varpi-\gamma|\varpi-1|=e^{\omega(z)}, $$

which implies that

2.5 $$ \varpi=\frac{e^{\omega(z)}-\gamma e^{-i\phi}}{1-\gamma e^{-i\phi }}. $$

From (2.4) and (2.5), we easily obtain

2.6 $$ \frac{z(Q_{\beta,p}^{\alpha}g(z))'}{Q_{\beta,p}^{\alpha}g(z)} =\frac{p-1-p\gamma e^{-i\phi}+e^{\omega(z)}}{1-\gamma e^{-i\phi}}. $$

Now, using (1.7) in (2.6) and making simple computations, we have

2.7 $$ \frac{Q_{\beta,p}^{\alpha-1} g(z)}{Q_{\beta,p}^{\alpha}g(z)} =\frac{(\alpha+\beta+p-2)-\gamma(\alpha+\beta+p-1)e^{-i\phi}+e^{\omega (z)}}{(\alpha+\beta+p-1)(1-\gamma e^{-i\phi})}, $$

which, by virtue of (2.3), yields the inequality

2.8 $$ \bigl\vert Q_{\beta,p}^{\alpha}g(z) \bigr\vert \leq \frac{(1+\gamma)(\alpha+\beta+p-1)}{ \vert \alpha+\beta+p-2 \vert -\gamma(\alpha+\beta+p-1)-e^{ \vert z \vert }} \bigl\vert Q_{\beta,p}^{\alpha-1} g(z) \bigr\vert . $$

Again, because $Q_{\beta,p}^{\alpha}f(z)$ is majorized by $Q_{\beta ,p}^{\alpha}g(z)$ in $\mathbb{U}$, so we find from (1.2) that

2.9 $$ Q_{\beta,p}^{\alpha}f(z)=\varphi(z)Q_{\beta,p}^{\alpha}g(z). $$

Differentiating (2.9) on both sides with respect to *z* and multiplying by *z*, we obtain

2.10 $$ z \bigl(Q_{\beta,p}^{\alpha}f(z) \bigr)'=z \varphi'(z)Q_{\beta,p}^{\alpha}g(z) +z\varphi(z) \bigl(Q_{\beta,p}^{\alpha}g(z) \bigr)'. $$

By using (1.7) in (2.10), together with (2.9), we have

2.11 $$ Q_{\beta,p}^{\alpha-1}f(z)=\frac{1}{\alpha+\beta+p-1}z\varphi '(z)Q_{\beta,p}^{\alpha}g(z) +\varphi(z)Q_{\beta,p}^{\alpha-1}g(z). $$

On the other hand, noticing that the Schwarz function *φ* satisfies the inequality (see, e.g., Nehari [24])

2.12 $$ \bigl\vert \varphi'(z) \bigr\vert \leq\frac{1-|\varphi(z)|^{2}}{1-|z|^{2}}\quad (z\in\mathbb {U}), $$

and in terms of (2.8) and (2.12) in (2.11), we get

$$\bigl\vert Q_{\beta,p}^{\alpha-1}f(z) \bigr\vert \leq \biggl[ \bigl\vert \varphi(z) \bigr\vert +\frac{|z|(1+\gamma)(1-|\varphi(z)|^{2})}{(1-|z|^{2}) (|\alpha+\beta+p-2|-\gamma(\alpha+\beta+p-1)-e^{|z|} )} \biggr] \bigl\vert Q_{\beta,p}^{\alpha-1}g(z) \bigr\vert , $$

which, by taking

$$|z|=r,\qquad \bigl\vert \varphi(z) \bigr\vert =\rho \quad (0\leq\rho\leq1), $$

reduces to the inequality

$$\bigl\vert Q_{\beta,p}^{\alpha-1}f(z) \bigr\vert \leq \Phi_{1}(r,\rho) \bigl\vert Q_{\beta,p}^{\alpha-1}g(z) \bigr\vert , $$

where

$$\Phi_{1}(r,\rho)=\frac{r(1+\gamma)(1-\rho^{2})}{(1-r^{2}) [|\alpha+\beta+p-2|-\gamma(\alpha+\beta+p-1)-e^{r} ]}+\rho. $$

In order to determine $r_{1}$, we must choose

$$\begin{aligned} r_{1} =&\max \bigl\{ r\in[0,1): \Phi_{1}(r,\rho)\leq1, \forall\rho\in [0,1] \bigr\} \\ =&\max \bigl\{ r\in[0,1): \Psi_{1}(r,\rho)\geq0, \forall\rho\in[0,1] \bigr\} , \end{aligned}$$

where

$$\Psi_{1}(r,\rho)=\bigl(1-r^{2}\bigr) \bigl[ \vert \alpha+ \beta+p-2 \vert -\gamma(\alpha+\beta+p-1)-e^{r} \bigr]-r(1+\gamma) (1+\rho). $$

Obviously, for $\rho=1$, the function $\Psi_{1}(r,\rho)$ takes its minimum value, namely

$$\min \bigl\{ \Psi_{1}(r,\rho): \rho\in[0,1] \bigr\} = \Psi_{1}(r,1):=\psi_{1}(r), $$

where

$$\psi_{1}(r)=\bigl(1-r^{2}\bigr) \bigl[ \vert \alpha+ \beta+p-2 \vert -\gamma(\alpha+\beta+p-1)-e^{r} \bigr]-2r(1+\gamma). $$

Further, because $\psi_{1}(0)=|\alpha+\beta+p-2|>\gamma(\alpha+\beta +p-1)+e$ and $\psi_{1}(1)=-2(1+\gamma)<0$, so there exists $r_{1}$ such that $\psi_{1}(r)\geq0$ for all $r\in[0,r_{1}]$, where $r_{1}=r_{1}(p,\alpha ,\beta,\gamma)$ is the smallest positive root of equation (2.1). This completes the proof of Theorem 2.1. □

## Majorization problem for the class $N_{\beta}^{\alpha}(p,\theta )$

Next, we discuss majorization property for the class $N_{\beta}^{\alpha }(p,\theta)$.

### Theorem 3.1

*Let the function* $f\in\mathcal{A}_{p}$ *and assume that* $g\in N_{\beta}^{\alpha}(p,\theta)$ *with* $|\alpha+\beta-1|\geq|\tan \theta||\alpha+\beta|+e$. *If* $Q_{\beta,p}^{\alpha}f(z)$ *is majorized by* $Q_{\beta,p}^{\alpha}g(z)$ *in* $\mathbb{U}$, *that is*,

$$Q_{\beta,p}^{\alpha}f(z)\ll Q_{\beta,p}^{\alpha}g(z) \quad (z\in\mathbb{U}), $$

*then*, *for* $|z|\leq r_{2}$, *we have*

3.1 $$ \bigl\vert Q_{\beta,p}^{\alpha-1}f(z) \bigr\vert \leq \bigl\vert Q_{\beta,p}^{\alpha-1}g(z) \bigr\vert , $$

*where* $r_{2}=r_{2}(\alpha,\beta,\theta)$ *is the smallest positive root of the equation*

3.2 $$\begin{aligned}& r^{2}e^{r}-\bigl[ \vert \alpha+\beta-1 \vert - \vert \tan\theta \vert \vert \alpha+\beta \vert \bigr]r^{2} -e^{r}-2 \vert \sec\theta \vert r+ \vert \alpha+\beta-1 \vert - \vert \tan\theta \vert \vert \alpha+\beta \vert =0 \\& \quad \biggl(\alpha\geq0; \beta>-1; -\frac{\pi}{2}< \theta< \frac{\pi}{2}\biggr). \end{aligned}$$

### Proof

Because $g\in N_{\beta}^{\alpha}(p,\theta)$, so from (1.10) we show that

3.3 $$ e^{i\theta} \biggl(\frac{z(Q_{\beta,p}^{\alpha}g(z))'}{Q_{\beta,p}^{\alpha}g(z)} \biggr)= e^{\omega(z)}\cos\theta+i \sin\theta, $$

where $\omega(z)$ is defined as (2.3).

From (3.3) it follows that

3.4 $$ \frac{z(Q_{\beta,p}^{\alpha}g(z))'}{Q_{\beta,p}^{\alpha}g(z)} =\frac{e^{\omega(z)}+i\tan\theta}{1+i\tan\theta}. $$

Now, putting (1.7) in (3.4) and making some calculations, we get

$$\frac{Q_{\beta,p}^{\alpha-1} g(z)}{Q_{\beta,p}^{\alpha}g(z)} =\frac{(\alpha+\beta-1)+i\tan\theta(\alpha+\beta)+e^{\omega (z)}}{(1+i\tan\theta)(\alpha+\beta+p-1)}, $$

which, using (2.3), becomes the inequality

3.5 $$ \bigl\vert Q_{\beta,p}^{\alpha}g(z) \bigr\vert \leq \frac{ \vert \sec\theta \vert (\alpha+\beta+p-1)}{ \vert \alpha+\beta-1 \vert - \vert \tan\theta \vert \vert \alpha+\beta \vert -e^{ \vert z \vert }} \bigl\vert Q_{\beta,p}^{\alpha-1} g(z) \bigr\vert . $$

Next, in view of (2.12) as well as (3.5) in (2.11), and just as the proof of Theorem 2.1, we have

$$\bigl\vert Q_{\beta,p}^{\alpha-1}f(z) \bigr\vert \leq \biggl[ \bigl\vert \varphi(z) \bigr\vert +\frac{ \vert z \vert \vert \sec\theta \vert (1- \vert \varphi(z) \vert ^{2})}{(1- \vert z \vert ^{2}) ( \vert \alpha+\beta-1 \vert - \vert \tan\theta \vert \vert \alpha+\beta \vert -e^{ \vert z \vert } )} \biggr] \bigl\vert Q_{\beta,p}^{\alpha-1} g(z) \bigr\vert , $$

which, by setting

$$|z|=r,\qquad \bigl\vert \varphi(z) \bigr\vert =\rho\quad (0\leq\rho\leq1), $$

reduces to the inequality

3.6 $$ \bigl\vert Q_{\beta,p}^{\alpha-1}f(z) \bigr\vert \leq \frac{\Phi_{2}(\rho)}{(1-r^{2}) [ \vert \alpha+\beta-1 \vert - \vert \tan\theta \vert \vert \alpha+\beta \vert -e^{r} ]} \bigl\vert Q_{\beta ,p}^{\alpha-1}g(z) \bigr\vert , $$

where the function $\Phi_{2}(\rho)$ given by

$$\Phi_{2}(\rho)=-r \vert \sec\theta \vert \rho^{2}+ \bigl(1-r^{2}\bigr) \bigl[ \vert \alpha+\beta-1 \vert - \vert \tan \theta \vert \vert \alpha+\beta \vert -e^{r} \bigr]\rho +r \vert \sec\theta \vert $$

takes its maximum value at $\rho=1$ with $r_{2}=r_{2}(p,\alpha,\beta,\theta )$ defined by (3.2). Furthermore, if $0\leq\sigma\leq r_{2}(p,\alpha,\beta,\theta)$, then the function

$$\Psi_{2}(\rho)=-\sigma \vert \sec\theta \vert \rho^{2}+ \bigl(1-\sigma^{2}\bigr) \bigl[ \vert \alpha+\beta-1 \vert - \vert \tan\theta \vert \vert \alpha+\beta \vert -e^{\sigma} \bigr]\rho + \sigma \vert \sec\theta \vert $$

increases on the interval $0\leq\rho\leq1$, therefore

$$\Psi_{2}(\rho)\leq\Psi_{2}(1)=\bigl(1-\sigma^{2} \bigr) \bigl[ \vert \alpha+\beta-1 \vert - \vert \tan\theta \vert \vert \alpha+\beta \vert -e^{\sigma} \bigr]\quad \bigl(0\leq\sigma\leq r_{2}(p,\alpha,\beta ,\theta)\bigr). $$

Hence, from this fact and (3.6), we conclude that inequality (3.1) holds true for $|z|\leq r_{2}$, where $r_{2}=r_{2}(p,\alpha ,\beta,\theta)$ is given by (3.2). We complete the proof of Theorem 3.1. □

## Some corollaries

As a special case of Theorem 2.1, when $p=1$, we get the following result.

### Corollary 4.1

*Let the function* $f\in\mathcal{A}$ *and assume that* $g\in M_{\beta}^{\alpha}(\gamma)$ *with* $|\alpha+\beta-1|\geq\gamma (\alpha+\beta)+e$. *If* $Q_{\beta}^{\alpha}f(z)$ *is majorized by* $Q_{\beta}^{\alpha}g(z)$ *in* $\mathbb{U}$, *then*, *for* $|z|\leq r_{3}$, *we have*

$$\bigl\vert Q_{\beta}^{\alpha-1}f(z) \bigr\vert \leq \bigl\vert Q_{\beta}^{\alpha-1}g(z) \bigr\vert , $$

*where* $r_{3}:=r_{1}(1,\alpha,\beta,\gamma)$ *is the smallest positive root of the equation*

$$\begin{aligned}& r^{2}e^{r}- \bigl[ \vert \alpha+\beta-1 \vert -\gamma( \alpha+\beta) \bigr]r^{2} -e^{r}-2(1+\gamma)r+ \vert \alpha+\beta-1 \vert -\gamma(\alpha+\beta)=0 \\& \quad (\alpha\geq 0; \beta>-1; \gamma\geq0). \end{aligned}$$

Setting $\gamma=0$ in Theorem 2.1, we obtain the following corollary.

### Corollary 4.2

*Let the function* $f\in\mathcal{A}_{p}$ *and assume that* $g\in M_{\beta}^{\alpha}(p)$ *with* $|\alpha+\beta+p-2|\geq e$. *If* $Q_{\beta,p}^{\alpha}f(z)$ *is majorized by* $Q_{\beta,p}^{\alpha}g(z)$ *in* $\mathbb{U}$, *then*, *for* $|z|\leq r_{4}$, *we have*

$$\bigl\vert Q_{\beta,p}^{\alpha-1}f(z) \bigr\vert \leq \bigl\vert Q_{\beta,p}^{\alpha-1}g(z) \bigr\vert , $$

*where* $r_{4}:=r_{1}(p,\alpha,\beta,0)$ *is the smallest positive root of the equation*

$$r^{2}e^{r}- \vert \alpha+\beta+p-2 \vert r^{2} -e^{r}-2r+ \vert \alpha+\beta+p-2 \vert =0 \quad (p\in\mathbb{N}; \alpha\geq0; \beta>-1). $$

Taking $\theta=0$ in Theorem 3.1, we state the following corollary.

### Corollary 4.3

*Let the function* $f\in\mathcal{A}_{p}$ *and suppose that* $g\in N_{\beta}^{\alpha}(p)$ *with* $|\alpha+\beta-1|\geq e$. *If* $Q_{\beta,p}^{\alpha}f(z)$ *is majorized by* $Q_{\beta,p}^{\alpha}g(z)$ *in* $\mathbb{U}$, *then*, *for* $|z|\leq r_{5}$, *we have*

$$\bigl\vert Q_{\beta,p}^{\alpha-1}f(z) \bigr\vert \leq \bigl\vert Q_{\beta,p}^{\alpha-1}g(z) \bigr\vert , $$

*where* $r_{5}:=r_{2}(\alpha,\beta,0)$ *is the smallest positive root of the equation*

4.1 $$ r^{2}e^{r}- \vert \alpha+\beta-1 \vert r^{2}-e^{r}-2r+ \vert \alpha+\beta-1 \vert =0 \quad ( \alpha\geq0; \beta >-1). $$

## Conclusions

In this paper, we investigate the problems of majorization of the classes $M_{\beta}^{\alpha}(p,\gamma)$ and $N_{\beta}^{\alpha}(p,\theta )$ defined by the Liu–Owa integral operator $Q_{\beta,p}^{\alpha}$ given by (1.5), which are also related to an exponential function. The results obtained generalize and unify the theory of majorization in geometric function theory. In addition, we notice that, if we put $p=1$ and $\alpha=1$, $\beta=\delta$ in Theorems 2.1 and 3.1, as well as Corollaries 4.2 and 4.3 of this paper, respectively, then we easily get the corresponding majorization results for the Jung–Kim–Srivastava integral operator $Q_{\beta}^{\alpha}$ and the generalized Libera operator $J_{\delta,p}$ ($\delta>-p$; $p\in\mathbb{N}$), which are mentioned in the Introduction.

## References

[1] Altintas O., Ozkan O., Srivastava H.M. (2001). Majorization by starlike functions of complex order. Complex Var. Theory Appl..

[2] Altintas O., Srivastava H.M. (2001). Some majorization problems associated with *p*-valently starlike and convex functions of complex order. East Asian Math. J..

[3] Aouf M.K. (2008). Inequalities involving certain integral operator. J. Math. Inequal..

[4] Aouf M.K., Bulboacă T. (2010). Subclasses of multivalent functions involving the Liu–Owa operator. Quaest. Math..

[5] Aouf M.K., Bulboacă T. (2010). Subordination and superordination properties of multivalent functions defined by certain integral operator. J. Franklin Inst..

[6] Aouf M.K., Seoudy T.M. (2010). Some properties of a certain subclass of multivalent analytic functions involving the Liu–Owa operator. Comput. Math. Appl..

[7] Aouf M.K., Seoudy T.M. (2011). Some preserving subordination and superordination of analytic functions involving the Liu–Owa integral operator. Comput. Math. Appl..

[8] Aouf M.K., Seoudy T.M. (2011). On a certain subclass of multivalent analytic functions defined by the Liu–Owa operator. Bull. Belg. Math. Soc. Simon Stevin.

[9] Aouf M.K., Seoudy T.M. (2013). Some preserving subordination and superordination of the Liu–Owa integral operator. Complex Anal. Oper. Theory.

[10] Bernardi S.D. (1969). Convex and starlike univalent functions. Trans. Am. Math. Soc..

[11] Gao C.-Y., Yuan S.-M., Srivastava H.M. (2005). Some functional inequalities and inclusion relationships associated with certain families of integral operator. Comput. Math. Appl..

[12] Goodman A.W. (1983). Univalent Functions.

[13] Goswami P., Aouf M.K. (2010). Majorization properties for certain classes of analytic functions using the Salagean operator. Appl. Math. Lett..

[14] Goyal S.P., Goswami P. (2009). Majorization for certain classes of analytic functions defined by fractional derivatives. Appl. Math. Lett..

[15] Goyal S.P., Goswami P. (2012). Majorization for certain classes of meromorphic functions defined by integral operator. Ann. Univ. Mariae Curie-Skłodowska, Sect. A.

[16] Janowski W. (1973). Some extremal problems for certain families of analytic functions. Ann. Pol. Math..

[17] Jung I.B., Kim Y.C., Srivastava H.M. (1993). The Hardy space of analytic functions associated with certain one-parameter families of integral operators. J. Math. Anal. Appl..

[18] Li S.-H., Tang H., En A. (2013). Majorization properties for certain new classes of analytic functions using the Salagean operator. J. Inequal. Appl..

[19] Libera R.J. (1965). Some classes of regular univalent functions. Proc. Am. Math. Soc..

[20] Liu J.-L., Owa S. (2004). Properties of certain integral operators. Int. J. Math. Math. Sci..

[21] Ma W., Minda D. (1991). An internal geometric characterization of strongly starlike functions. Ann. Univ. Mariae Curie-Skłodowska, Sect. A.

[22] MacGregor T.H. (1967). Majorization by univalent functions. Duke Math. J..

[23] Mendiratta R., Nagpal S., Ravichandran V. (2015). On a subclass of strongly starlike functions associated with exponential function. Bull. Malays. Math. Sci. Soc..

[24] Nehari Z. (1955). Conformal Mapping.

[25] Owa S., Srivastava H.M. (1986). Some applications of the generalized Libera integral operator. Proc. Jpn. Acad., Ser. A, Math. Sci..

[26] Panigrahi T., El-Ashwah R. (2017). Majorization for subclasses of multivalent meromorphic functions defined through iterations and combinations of the Liu–Srivastava operator and a meromorphic analogue of the Cho–Kwon–Srivastava operator. Filomat.

[27] Prajapat J.K., Aouf M.K. (2012). Majorization problem for certain class of *p*-valently analytic functions defined by generalized fractional differintegral operator. Comput. Math. Appl..

[28] Roberston M.S. (1970). Quasi-subordination and coefficient conjectures. Bull. Am. Math. Soc..

[29] Srivastava H.M., Owa S. (1992). Current Topics in Analytic Function Theory.

[30] Tang H., Aouf M.K., Deng G.-T. (2015). Majorization problems for certain subclasses of meromorphic multivalent functions associated with the Liu–Srivastava operator. Filomat.

[31] Tang H., Deng G.-T., Li S.-H. (2013). Majorization properties for certain classes of analytic functions involving a generalized differential operator. J. Math. Res. Appl..

[32] Tang H., Deng G.-T., Li S.-H. (2015). Double subordination preserving properties for the Liu–Owa operator. J. Math..

[33] Tang H., Li S.-H., Deng G.-T. (2014). Majorization properties for a new subclass of *θ*-spiral functions of order *γ*. Math. Slovaca.

